# Dentomaxillofacial imaging with panoramic views and cone beam CT

**DOI:** 10.1007/s13244-014-0379-4

**Published:** 2015-01-10

**Authors:** Anni Suomalainen, Elmira Pakbaznejad Esmaeili, Soraya Robinson

**Affiliations:** 1 Department of Radiology, Helsinki University Central Hospital and University of Helsinki, Helsinki, Finland; 2 Diagnose Zentrum Urania, Laurenzerberg 2a, 1010 Vienna, Austria; 3 Department of Radiology, Helsinki University Central Hospital, P.O. Box 263, 00029 HUS Helsinki, Finland

**Keywords:** Panoramic radiography, Cone beam computed tomography, Dentomaxillofacial imaging, Diagnosis, Radiation dose

## Abstract

Panoramic and intraoral radiographs are the basic imaging modalities used in dentistry. Often they are the only imaging techniques required for delineation of dental anatomy or pathology. Panoramic radiography produces a single image of the maxilla, mandible, teeth, temporomandibular joints and maxillary sinuses. During the exposure the x-ray source and detector rotate synchronously around the patient producing a curved surface tomography. It can be supplemented with intraoral radiographs. However, these techniques give only a two-dimensional view of complicated three-dimensional (3D) structures. As in the other fields of imaging also dentomaxillofacial imaging has moved towards 3D imaging. Since the late 1990s cone beam computed tomography (CBCT) devices have been designed specifically for dentomaxillofacial imaging, allowing accurate 3D imaging of hard tissues with a lower radiation dose, lower cost and easier availability for dentists when compared with multislice CT. Panoramic and intraoral radiographies are still the basic imaging methods in dentistry. CBCT should be used in more demanding cases. In this review the anatomy with the panoramic view will be presented as well as the benefits of the CBCT technique in comparison to the panoramic technique with some examples. Also the basics as well as common errors and pitfalls of these techniques will be discussed.

*Teaching Points*

• *Panoramic and intraoral radiographs are the basic imaging methods in dentomaxillofacial radiology*.

• *CBCT imaging allows accurate 3D imaging of hard tissues*.

• *CBCT offers lower costs and a* *smaller size and radiation dose compared with MSCT*.

• *The disadvantages of CBCT imaging are poor soft tissue contrast and artefacts*.

• *The Sedentexct project has developed evidence*-*based guidelines on the use of CBCT in dentistry*.

## Introduction

Intraoral and panoramic radiographs are the basic imaging techniques in dentomaxillofacial radiology, allowing two-dimensional (2D) imaging of oral hard tissues [1]. Often these imaging methods fulfil the requirements for dental imaging. However, these 2D, plain radiography methods have a limited capability in the evaluation of 3D relationships. As the technological advances in radiological imaging have led to the introduction of new methods in many fields of radiology, this also applies to dentomaxillofacial radiology. While multislice computed tomography (MSCT) imaging is a well-known 3D imaging method, cone beam CT (CBCT) is a fairly recent newcomer as these devices were introduced in dentomaxillofacial imaging in the late 1990s [2, 3]. Nowadays CBCT imaging is a widely used imaging method in dentomaxillofacial radiology, allowing accurate 3D imaging of hard tissue structures.

The benefits of CBCT are the lower cost, smaller size and smaller radiation dose compared with MSCT. In addition, these devices are more easily available for dentomaxillofacial examinations than MSCT devices. The disadvantages of CBCT imaging are poor soft tissue contrast and artefacts.

The first basic principles for the use of dental CBCT were introduced by both the American Academy of Oral and Maxillofacial Radiology and the European Academy of Dental and Maxillofacial Radiology [4, 5]. Soon after that, evidence-based guidelines for the use of CBCT in dental and maxillofacial radiology were prepared by the Sedentexct Project, i.e. European Commission guidelines [6]. In addition, the European Academy of Dental and Maxillofacial Radiology has prepared a position paper for basic training requirements for the use of dental CBCT by dentists [7].

## Panoramic tomography

### Technique and image formation

Panoramic tomography (PTG) provides a comprehensive 2D view of the jaws [8, 9]. Its broad coverage of maxillomandibular structures, low radiation dose (Table 1), relatively short exposure time, comfort and simplicity of the extraoral examination for experienced radiographers are seen as advantages of the method. Disadvantages are the lower image quality compared to intraoral radiographs, operator-dependence, geometric distortion such as unequal magnification and elongation, overlapping in the premolar region, superimposition of the cervical spine in the incisor region and presence of ghost images [8, 9, 11].

**Table 1:** Effective dose from conventional dental imaging techniques (i.e. intraoral, panoramic and cephalometric radiograph), cone beam computed tomography (*CBCT*) and multislice computed tomography (*MSCT*) in μSv. The range of the effective dose and median values (in parentheses) from dental CBCT in μSv (according to the Tables by EC 2012 [6]). For comparison, in Finland the annual radiation dose is approximately 3,200 μSv, i.e. approximately 10 μSv/day [10]

Imaging method	Effective dose [μSv]
Intraoral radiograph	<1.5
Panoramic radiograph	2.7–24.3
Cephalometric radiograph	<6
Dentoalveolar CBCT (FOV height <10 cm)	11–674 (61)
Craniofacial CBCT (FOV height >10 cm)	30–1,073 (87)
MSCT maxillo-mandibular	280–1,410

The PTG technique is based on the principle of narrow-beam rotational tomography where the X-ray beam is angled upward at approximately 8° [11] and uses linked motion of the x-ray tube head and receptor [9, 11, 12]. Because of the tomographic nature of the technique, only structures located within the tomographic plane are well delineated and those in front or behind that plane are blurred [9, 12]. This tomographic plane, also called the image layer (IL), is horseshoe shaped. Objects located inside the IL will appear wider and objects located in front of it will appear narrower. The central region of the IL is called the central plane (CP) of the image layer. Theoretically only objects located in this plane are depicted sharply and undistorted on the final image. Outside the CP of the IL, the discrepancy between the horizontal and vertical magnification is responsible for the distortion, the latter being smaller. Overlapping of the premolars cannot be avoided in the standard panoramic programme because of the anatomy of the jaws [9].

Distortion and overlapping are the reasons why the horizontal measurements are unreliable on PTG [9, 11, 12]. Some modern panoramic devices offer a multilayer panoramic programme, increasing the thickness of the focal area compared to traditional panoramic imaging. This decreases patient positioning errors and aids in difficult malocclusion cases.

### Image quality and errors

**Fig. 1:**
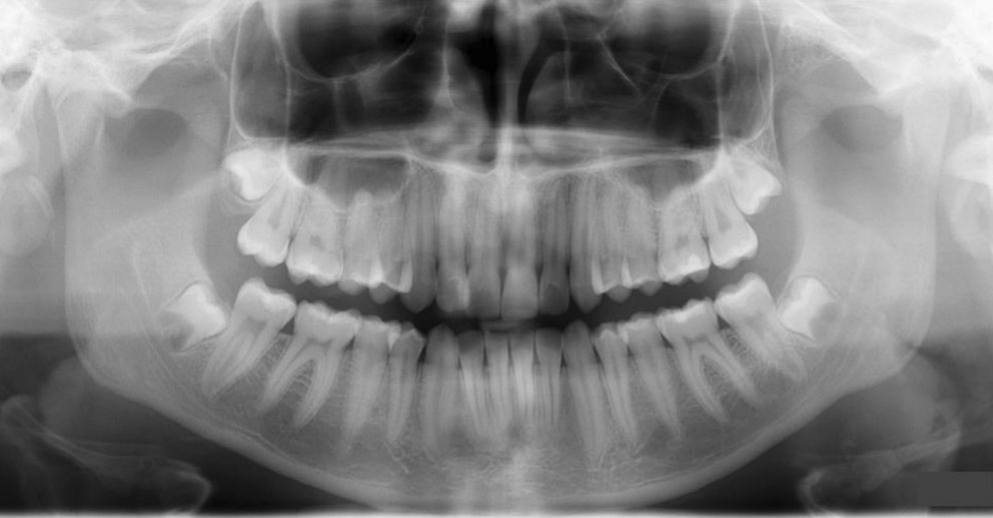
An optimal PTG without significant distortion or errors of a patient with developing wisdom teeth

An optimum PTG should display the jaws and their relative structures clearly without significant distortion or blurring [8, 12] (Fig. 1). Other ideal quality criteria include: equal magnification in the horizontal and vertical plane, the same mesiodistal dimension of the right and left molar teeth, uniform density all over the image and no evidence of artefacts. The hard palate should be imaged above the apices of the upper teeth. A light ghost shadow of the contralateral angle of the mandible and the cervical spine is accepted [11].

**Fig. 2:**
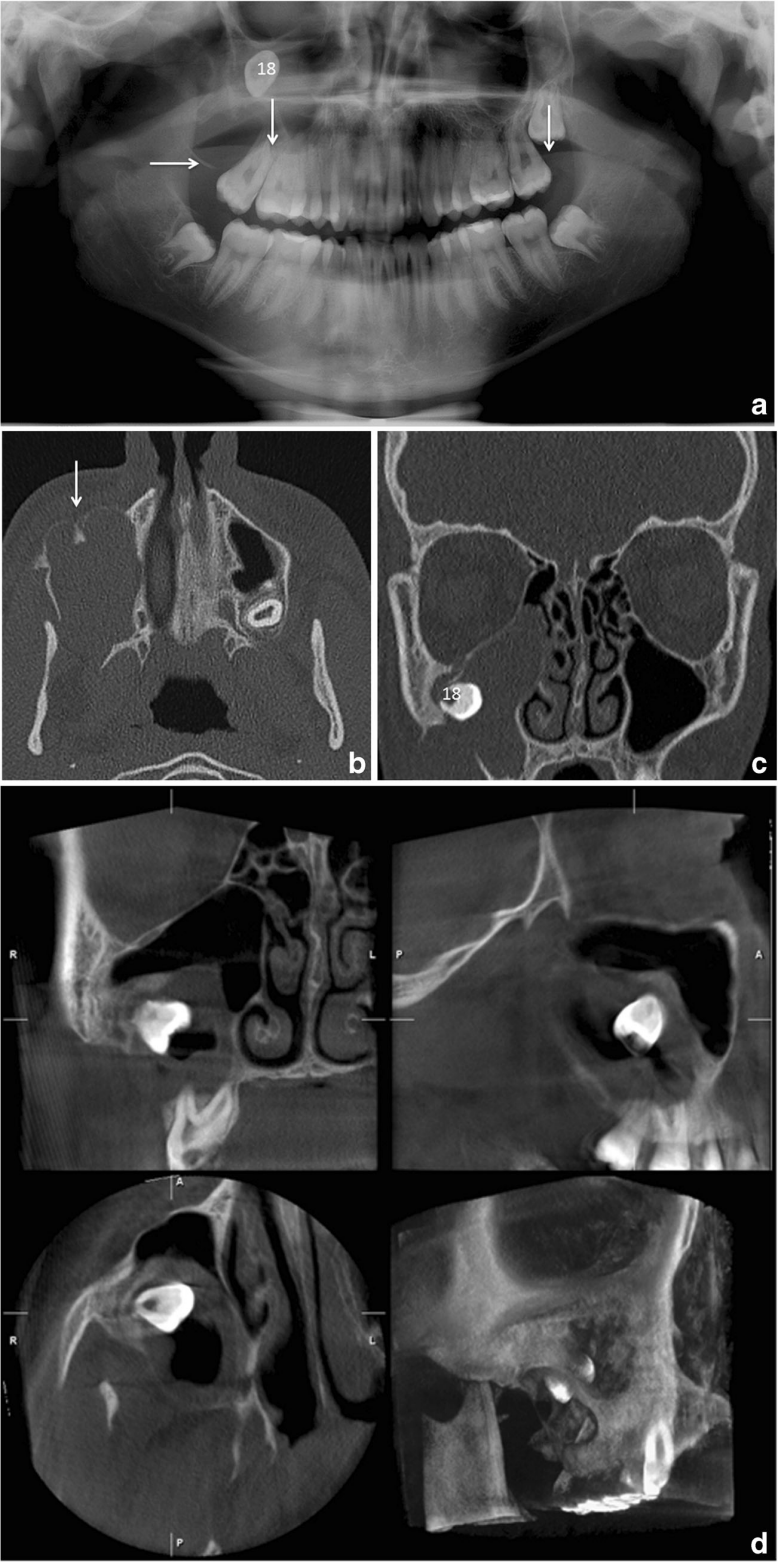
**a** PTG reveals cranial dislocation of d. 18 into the right maxillary sinus with a cystic lesion causing expansion of the sinus walls. Note the head positioning error in the PA plane; the patient has been positioned anterior to the image layer. Also the tongue (*vertical arrows*) is not against the palate. **b** Low dose MSCT axial and **c** coronal images show dislocated d. 18 surrounded by a large cystic lesion that causes expansion and perforation of the sinus wall. In the anterior wall of the sinus characteristic border scalloping of keratocystic odontogenic tumour (*KCOT*) is evident (*arrow*). **d** CBCT examination taken 5 months after the fenestration with concomitant biopsy of the lesion. The tumour was later operated

Common errors during PTG that affect the image quality include the following: (1) patient preparation errors such as failure to remove metallic detachable items in the area of the head and neck or inappropriate use of a thyroid shield during the exposure; (2) errors in selecting the correct exposure factors; (3) high sensitivity to positioning error. Head positioning error causes distortion in the horizontal plane more easily than in the vertical plane, especially in the anterior region of the jaws [9, 13]. If incisors are not biting edge to edge, the anterior teeth are displayed as wide or narrow (posteroanterior error) (Fig. 2). If the midsagittal plane is not symmetric with the mid light beam, the premolar/molar teeth will appear wider on the other side (horizontal error). The Frankfurt plane should be horizontal (vertical error) and the spine should be straight (ghosting shadow error). The patient’s shoulder should not touch the cassette holder during its movement and the tongue should be placed against the palate to avoid a disturbing rim of air (Fig. 2). The lips should be closed around the bite block (air shadow error). Patient movement would lead to distortion [11, 13].

### Anatomy and interpretation

In addition to understanding the processes of image formation and how the structures will appear on a PTG, deep knowledge of the complex normal maxillofacial hard and soft tissue anatomy, range of normal appearances and features of pathological conditions is necessary for proper analysis and accurate interpretation of a PTG [9, 11, 14]. In PTG projection curved maxillomandibular structures are “spread out” [14]. A PTG consists of three images superimposed on one: a posteroanterior image of the structures located mesially to the canines and two lateral images of the structures located distally to the canines [9].

**Fig. 3:**
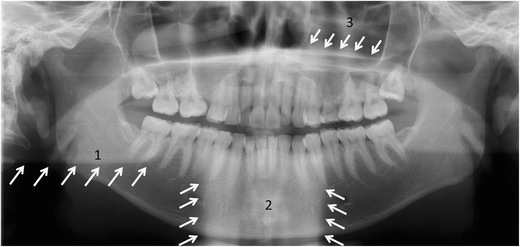
Main anatomical ghost shadows in a PTG: 1, contralateral angle and body of the mandible; 2, cervical spine; 3, contralateral hard palate. Note missing or extracted dd. 15, 38, 48; persistent d. 65 and peg-shaped d. 22. Polypoid swelling or retention cyst in the alveolar recess of the right maxillary sinus

Normal anatomical opacities on PTG in general are divided into real densities from structures within the IL between the rotation centre of the beam and the image receptor and ghost shadows (Fig. 3), which form from those hard tissues located on the opposite side of the IL between the X-ray source and the centre of rotation. The latter are always magnified and distorted, and they appear either over the midline or on the opposite side of the image in reversed configuration more cranially than the structures that caused them [9, 11].

**Fig. 4:**
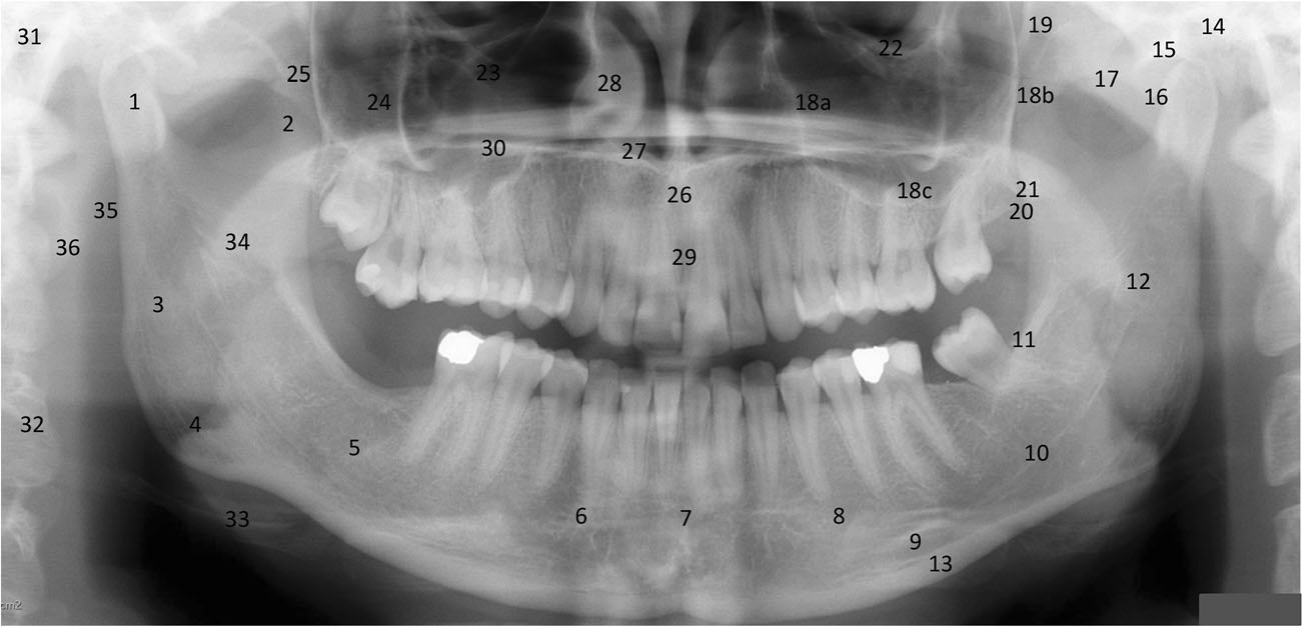
Main real hard tissue, soft tissue and air shadows in a PTG: 1, condylar process; 2, coronoid processes; 3, ramus; 4, angle; 5, body; 6, parasymphysis area; 7, symphysis area; 8, foramen mentale; 9, submandibular fossa; 10, mandibular canal; 11, linea oblique externa; 12, foramen mandibulae; 13, cortical border of the mandible; 14, glenoid fossa; 15, articular surfaces of the temporal bone; 16, articular eminence; 17, zygomatic arch; 18, a, b, c anterior and posterior cortical boundaries and floor of the maxillary sinus; 19, pterygomaxillary fissure; 20, maxillary tuberosity; 21, hamulus; 22, orbital rim; 23, infraorbital canal; 24, body of zygoma; 25, temporozygomatic fissure; 26, anterior nasal spine; 27, floor of the nasal cavity; 28, inferior nasal concha; 29, foramen incisivum; 30, hard palate; 31, external auditory meatus; 32, body of the cervical vertebra; 33, hyoid bone; 34, soft palate; 35, nasopharyngeal air shadow; 36, ear lobe

Detailed knowledge of the anatomy is the key to recognising pathology. The main anatomical shadows and airway shadows on a panoramic tomograph are marked in Fig. 4. A systematic approach helps analysis and takes place with the assessment of following:

1. Entire radiograph: The entire radiograph must be overviewed to assess the developmental stage of the dentition, developmental stage of a single tooth and location of each tooth/tooth follicle, tooth eruption, and possible supernumerary and missing teeth (Fig. 5) [15].

**Fig. 5:**
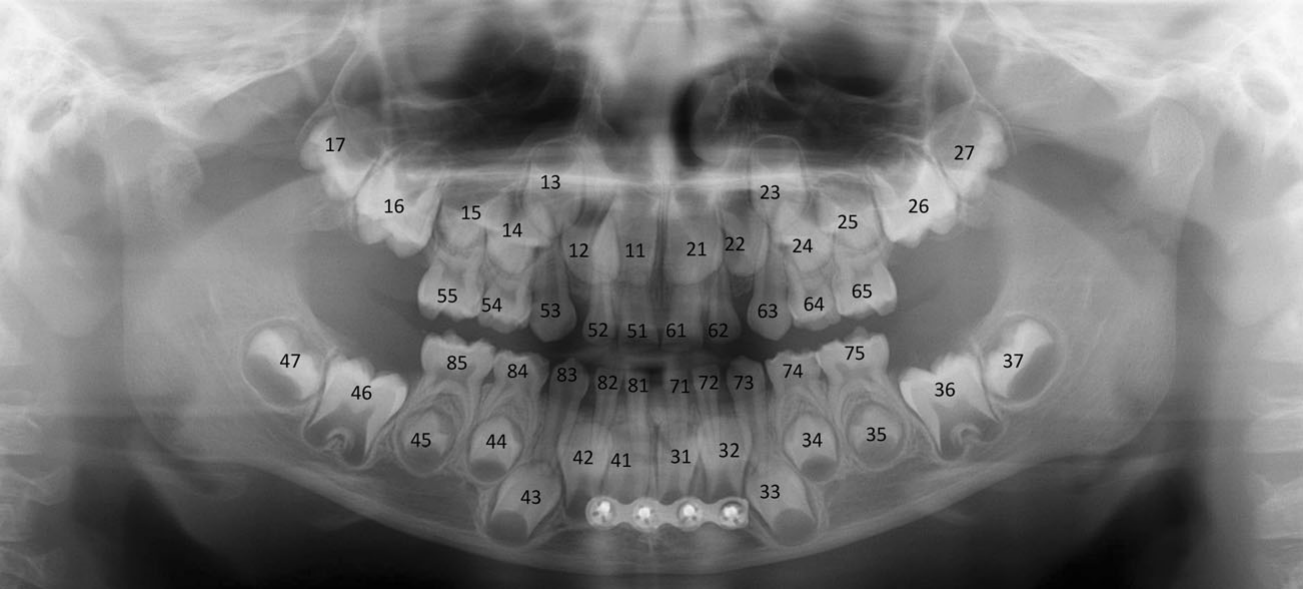
A PTG demonstrating deciduous dentition with all unerupted permanent teeth and their follicles (except third molars) in a 5-year- and 9-month-old male. The image was taken for postoperative control of the mandibular fractures: a plate in the symphysis area and conservative treatment with the condylar fractures
2. Teeth and their relative structures (apical and periodontal tissues): Teeth can be numbered using three internationally accepted systems. We present the Federation Dentaire Internationale (FDI) two-digit tooth numbering system, which is used worldwide. Each permanent tooth is given one number representing its quadrant (1, right maxilla; 2, left maxilla; 3, left mandible; 4, right mandible) and a second number from 1 to 8, 1 being the central incisor and 8 the wisdom tooth [16]. For primary teeth, the sequence of numbers goes 5, 6, 7 and 8 for the teeth in the upper right, upper left, lower left and lower right respectively (Fig. 5). Because of the 2D view in a PTG, we can only observe the mesial surface (near/toward to the midsagittal plane/midline) and the distal surface (farther/away from the midline/midsagittal plane) of the crowns (Fig. 6). The palatal (near the palate), lingual (near the tongue in the lower jaws) and buccal surface (near the cheeks) cannot be assessed reliably on a PTG.

**Fig. 6:**
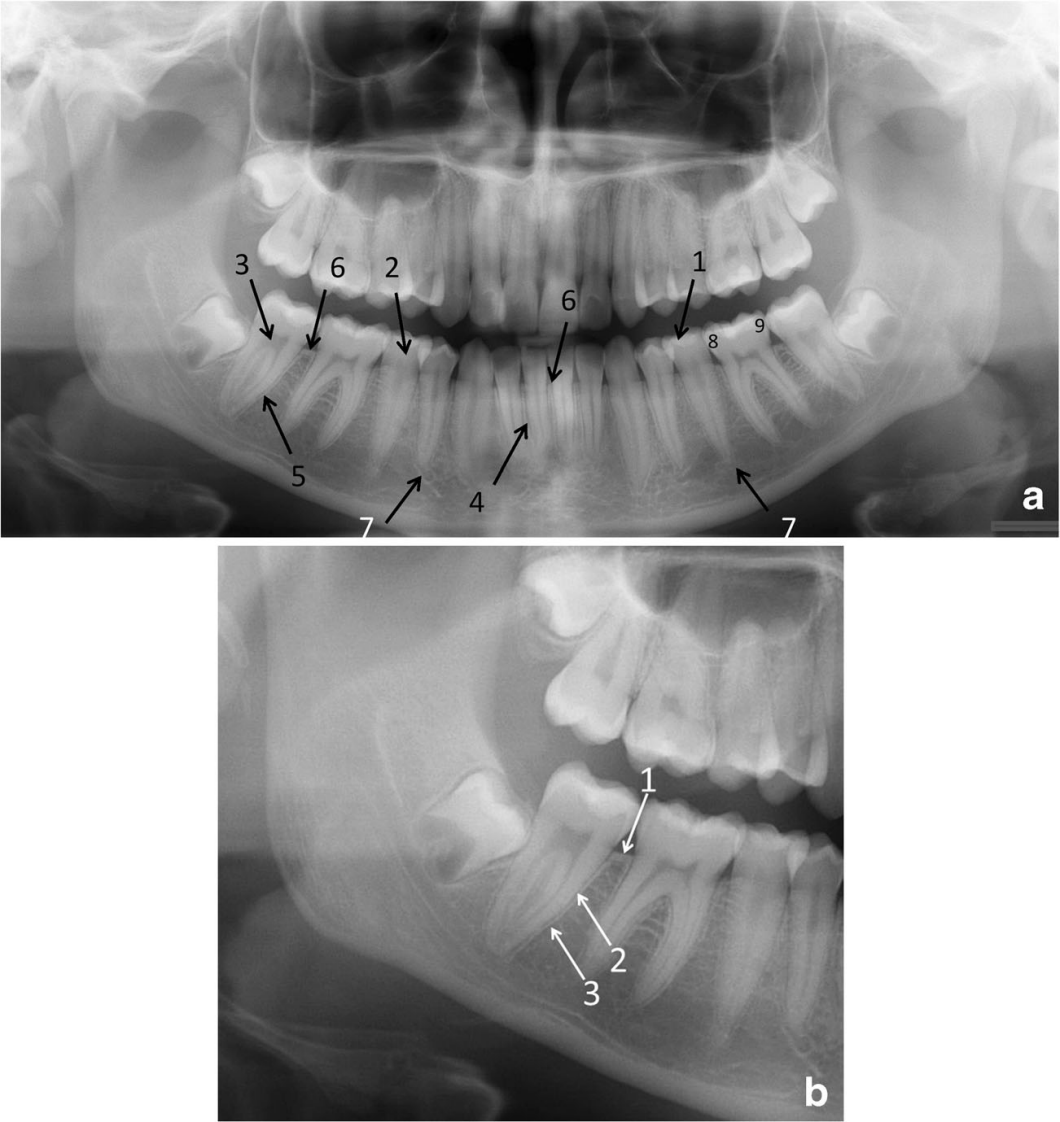
**a** Fine anatomical structures of teeth and their supporting structures in a PTG and **b** in a part of a PTG image: (a) 1, enamel; 2, dentine; 3, pulp chamber; 4, periodontal ligament space (fine radiolucent line around the root); 5, lamina dura (fine radiopaque line around the root); 6, crestal margin; 7, periapical area; 8, mesial side of the tooth; 9, distal side of the tooth. (b) 1, crestal margin; 2, periodontal ligament space; 3, lamina dura
  Each tooth should be evaluated for the presence of caries, restorations, number and morphology of the root, root filling and resorptions. The apical and periodontal tissue will be checked for the integrity, continuity, width and thickness of the radiolucent line around the root (periodontal ligament space) and the radiopaque line (lamina dura) (Fig. 6), any associated radiolucent or radiopaque finding next to the apex of the tooth, the pattern and density of the surrounding bone, the level and quality of the crestal bone, integrity of the corticated crestal margin, signs of horizontal or vertical bone loss, any calculus deposits and signs of bone loss in the furcation area (Fig. 7). However, supplementary intraoral radiographs are often indicated for example for the apical and the periodontal tissue evaluation and for caries diagnostics.

**Fig. 7:**
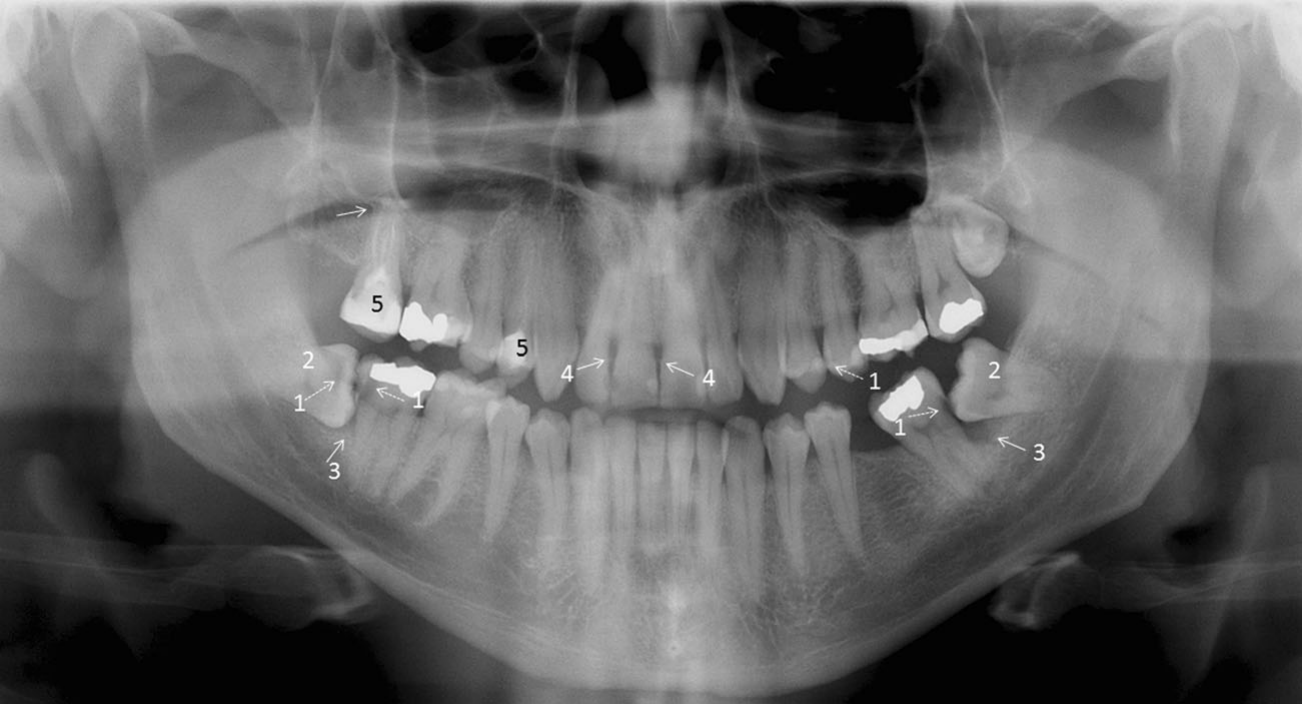
A PTG demonstrating frequent pathological conditions of teeth and periodontal (bony supportive) structures: 1. Carious lesions [dentine caries d. 37 distocervically, d. 47 distocervially, 48 occlusally and enamel caries d. 25 mesially (*dashed arrows*)]; bitewing radiography would be helpful for caries diagnostics. 2. Partially, mesioangulary erupted dd. 38, 48 and chronic pericoronitis with sclerosing osteitis next to d. 38 crown. 3. Alveolar bone loss in the region of dd. 37 and 47 distally associated with partially erupted dd. 38, 48 (*arrows*). 4. Calculus deposit best visible in dd. 12, 21 (*arrows*). 5. Dd. 17, 14 with inadequate root fillings; apical periodontitis of d. 17 (*arrow*) cannot be excluded and a periapical radiography is indicated. Also opacity of the floor of the right maxillary sinus is suspected
3. During the systematic approach of a PTG, the cortical border of the mandible must be followed and assessed for its integrity around the entire bone [15]. Also the condition of the outline of the mandibular canal should be assessed to exclude pathology [9]. It has to be remembered that only severe changes of the condylar head and glenoid fossa can be evaluated on a PTG (Fig. 8) [17]. Further investigation of the temporomandibular joint (TMJ) and evaluation of articular surfaces requires image modalities such as (CB)CT (for the evaluation of hard tissues) and magnetic resonance imaging (MRI) (for the evaluation of soft tissues) [18, 19].

**Fig. 8:**
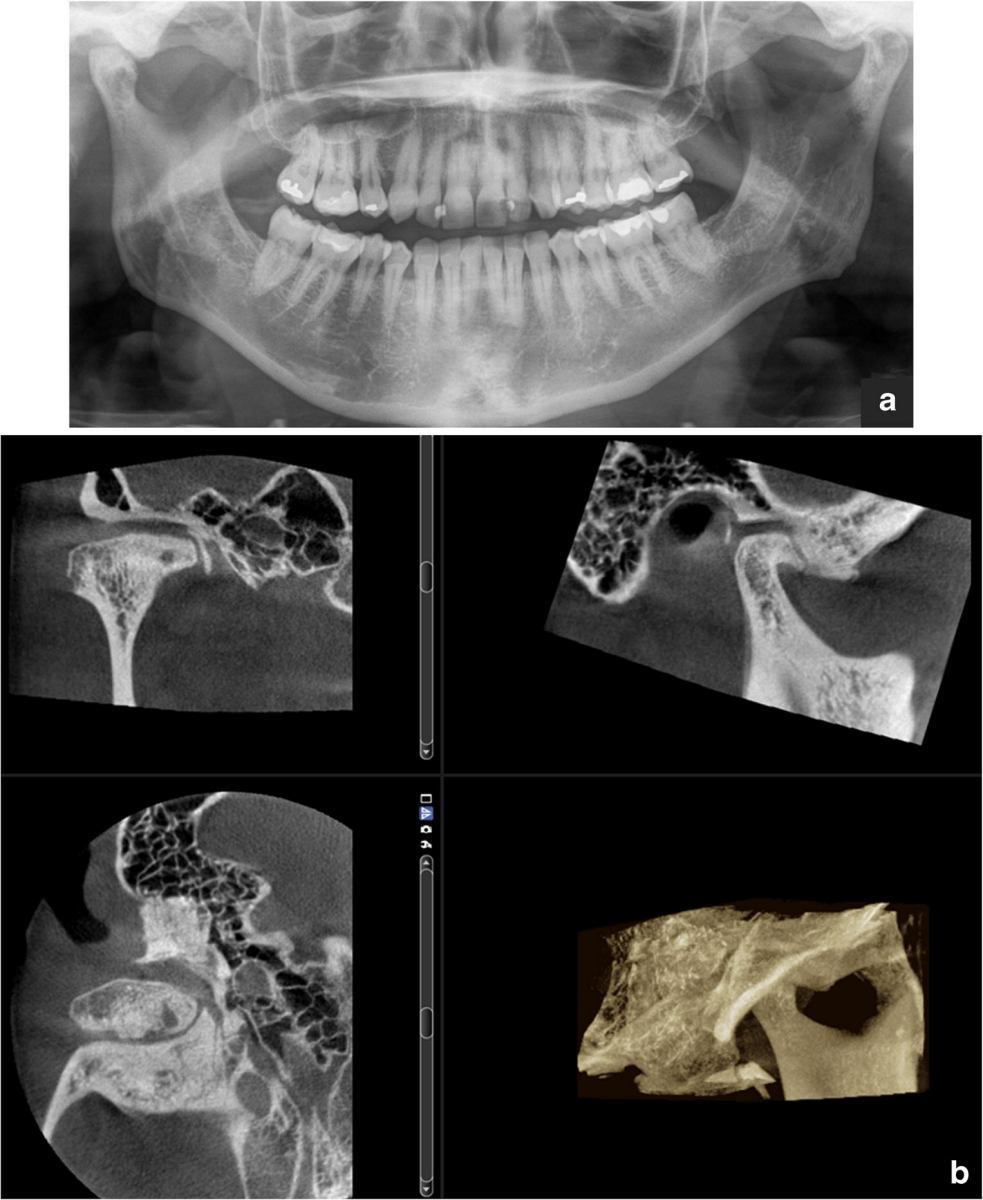
**a** Osteoarthrotic: flattening, osteophyte and subcortical cyst of the condyle, sclerosis in both the condyle and fossa, joint space narrowing: findings in a PTG visualised more clearly in **b** the CBCT (*right TMJ*)
4. In the midfacial region the outlines of the maxilla and aeration of the sinuses should be evaluated to exclude pathology in these areas (Fig. 2). However, PTG has limited value for evaluation of sinus or midfacial pathology as only portions of these structures that are within the IL will be demonstrated [20]. The whole maxillomandibular bone must be evaluated for any signs of pathological conditions [9].

Whenever PTG and intraoral imaging cannot provide the information needed; (CB)CT examination is considered in more complicated cases when hard tissue imaging is indicated.

## CBCT

### CBCT devices and technical basis of CBCT

**Fig. 9:**
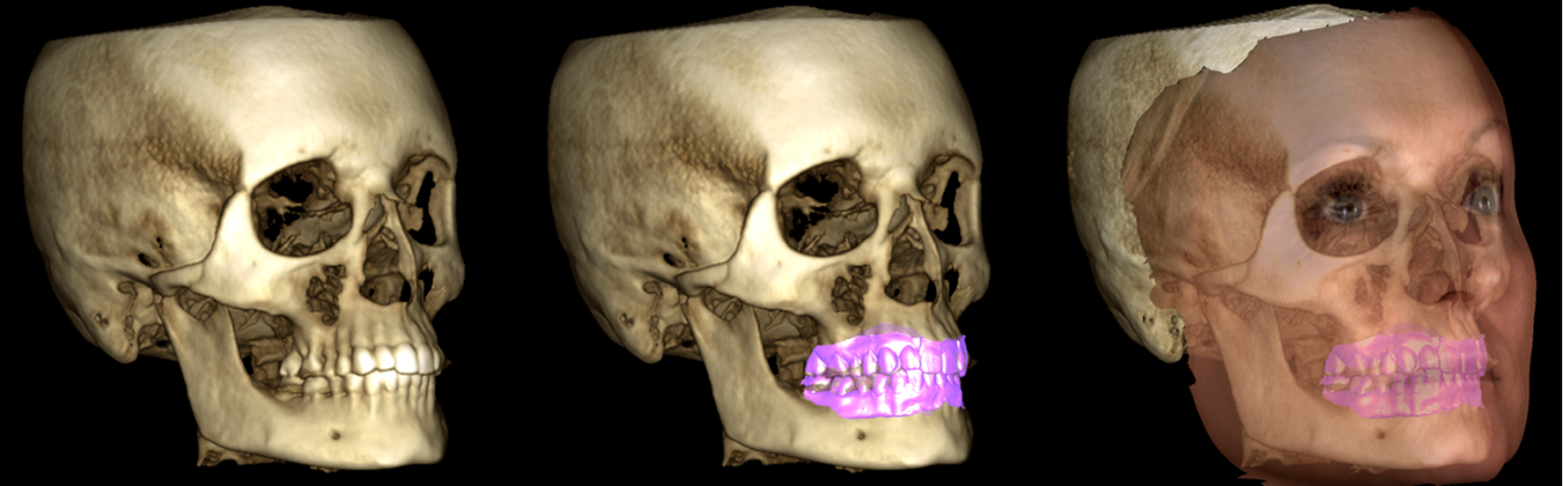
CBCT examination combined with 3D photography. (Courtesy of the manufacturer. The image is shown with the patient's permission)

According to a recent review a total of 47 CBCT devices for dentomaxillofacial imaging by 20 companies were available when the focus was on the European market [21]. Some devices are multimodal imaging devices including 3D CBCT imaging, digital 2D panoramics and cephalometry in the same unit. Some manufacturers also offer 3D photography in addition to the previously mentioned imaging (Fig. 9).

Depending on the device used, the patient is in the standing, sitting or supine position during the CBCT examination. The height and diameter of the field of view (FOV) vary from small to large field examinations [21, 22]. In addition, in some devices one can stitch adjacent CBCT volumes, allowing larger FOVs [23]. Devices allowing variation of the FOV and resolution, thus making task-specific protocols, are indicated in dentomaxillofacial imaging [22].

CBCT imaging is accomplished using a rotating gantry to which an X-ray source and detector are fixed. Nearly all modern CBCT devices use a digital flat panel detector (FPD) instead of an image intensifier for image capture. CBCT scanners use a tightly collimated narrow cone-shaped X-ray beam [22]. Image data are recorded in a single gantry rotation (180–360°) when the x-ray source and 2D detector move synchronously around the patient’s head, which is stabilised with a head holder. The acquisition time of CBCT devices ranges roughly between 6 and 20 s [24]. Posteroanterior and lateral scout views can be used to determine the correct location of the imaging area and its use is recommended especially in small FOV examinations. However, it is important to notice that the positioning of the FOV using a single projection is prone to error because of the summing of attenuation structures in the depth direction and divergence of the beam [23].

During the exposure multiple sequential planar projection images of the FOV are acquired [22]. Some devices use continuous radiation exposure instead of pulsed X-ray beam exposure. When the basis projection images have been acquired, the CBCT unit reconstructs the primary projection frames to provide standard viewing displays of coronal, sagittal and axial images similar to the MSCT data display (Fig. 2) [22]. In addition a 3D reconstruction image is typically produced (Fig. 2). Also oblique planar, curved planar (e.g. panoramic reconstruction) and serial transplanar reformations (e.g. implant site assessment) can be reconstructed [25]. Any multiplanar image can be thickened by increasing the number of adjacent voxels included in the slice. The simplest technique is adding the absorption values of adjacent voxels producing a ray sum image (e.g. panoramic and cephalometric images). These ray sum images are without magnification and distortion. However, it is important to notice that metal artefacts in these reconstructed images caused for example by dental fillings are found—crown fillings causing fewer artefacts in the area of the alveolar process than pins in the roots or dental implants. Another thickening technique is maximum intensity projection (MIP) where only the highest voxel value within a particular thickness will be displayed, producing a pseudo-3D structure. This is useful for visualising the surface morphology. In addition 3D reconstruction can be produced where the entire thickness of the volumetric data set is used, applying more complicated algorithms [25].

### Radiation dose and image quality

The radiation doses from dental CBCT are generally higher than in conventional dental radiography (intraoral, panoramic, cephalometric radiography) but lower than in MSCT of the dental area (Table 1) [6]. The dose is dependent on equipment type and exposure settings, especially the FOV, exposure time (s), tube current (mA) and the energy/potential (kV) [26]. It is also dependent on the region being scanned because radiosensitive tissues, for example the salivary glands and thyroid gland, are irradiated differently in different examinations. Also, the use of lead shielding reduces the absorbed doses to the thyroid [27]. The personnel using a CBCT device must have appropriate knowledge of and training in patient radiation doses related to the specific device they are using.

The large variations in patient doses emphasise the importance of optimising imaging parameters in both CBCT and MSCT examinations. The radiation dose should be kept as low as possible following the ALARA (as low as reasonably achievable) principle and it should be in balance with image quality. Standards should be developed for the image quality and dose for different diagnostic tasks. At the present only a single diagnostic reference level (DRL) of 250 mGy cm^2^ for the placement of an upper first molar implant in adults is available [6]. In the future low-dose protocols on modern CBCT and MSCT equipment most probably bring the doses down significantly.

CBCT has excellent high-contrast resolution as a result of the small size—down to 0.076 mm—and the geometry of its isotropic voxels is equal in all three dimensions [22]. This produces sub-millimetre resolution often exceeding the highest grade MSCT [25]. One disadvantage of CBCT devices designed for dentomaxillofacial examinations is the poor soft tissue resolution. In this respect CBCT images are comparable to the bone window images of the MSCT examination. Poor soft tissue contrast is not usually a problem in dentomaxillofacial radiology because the main interest is generally hard tissues—teeth and bones. However, with a different equipment design the CBCT method is also applicable to the imaging of soft tissues [28] but then the radiation dose is the same or nearly the same as with MSCT examinations [29, 30].

Artefacts in CBCT as well as with MSCT can be physics based, patient related or scanner based [22, 31]. In addition to these artefacts also cone-beam-related artefacts are characteristic to CBCT [22]. The Feldkamp algorithm—the standard for image reconstruction in CBCT at the present—guarantees a high image quality in the central plane of the CBCT examination and the image quality degrades as a function of distance from that plane [24]. This must be taken into account especially with the large FOV examinations [24].

One typical cause for artefacts in the CBCT examination is patient motion and a sufficient fixation of the patient’s head during the imaging process is most important to avoid movement of the patient. In the CBCT examination the patient’s movements affect the quality of the entire volume data, whereas in MSCT only those slices during which movement occurred were affected. The smaller the voxel size is, the smaller the movement necessary to move the patient structures out of the “correct” voxels [24]. Movement artefacts typically present as double contours [24]. In the future, detector hardware will most probably enable faster detector read-out, thereby reducing imaging times and thus chances for patient movement [24].

Another typical cause for artefacts is metal including metal restorations, orthodontic appliances and dental implants [22]. Metal and windmill artefacts are generally reduced in CBCT compared to MSCT, especially for high-density metals [32]. In addition, for example, root canal filling materials cause artefacts. To minimise metal artefacts in both CBCT and MSCT, artefact suppression algorithms are used and will be further developed [24].

Artefacts also challenge the accurate conversion of density values into Hounsfield units (HU) and large FOV CBCT examinations are generally more affected by artefacts compared with small FOV CBCT examination [33]. Most CBCT devices have a good overall correlation with HU units, but large errors can be seen when using the grey values in a quantitative way [34]. Kyriakou et al. [35] have shown that when imaging a homogeneous water phantom, the HU units are not uniform over the entire cross section and they decline towards the edges.

### Clinical use of CBCT

The indications for the use of CBCT imaging in dentomaxillofacial radiology have been presented in the Sedentexct evidence-based guidelines [6]. The aim of the Sedentexct project was to develop comprehensive, evidence-based guidelines on the use of CBCT in dentistry, including the referral criteria, quality assurance guidelines and optimisation strategies. As with all imaging, we have to follow the ALARA principle and no routine use of CBCT is allowed. Patient history, clinical information and previous images have to be available before CBCT imaging. In addition, if 2D radiographs do not or are not to be expected to answer the diagnostic question and it is expected that CBCT adds new relevant information, the use of CBCT can be considered justified. A lot of research is going on in this field and thus these Sedentexct recommendations have to be reviewed and updated. This is particularly important for referral and even more for self-referral criteria. More research is needed particularly to show the possible benefits of the use of CBCT in the treatment outcome in comparison to conventional dental imaging methods—panoramic, intraoral and cephalometric radiography.

In the Sedentexct guidelines, CBCT use for developing dentition (localised applications to answer a specific question and generalised application for examination of the entire dento-facial region), restoring the dentition (dental caries diagnosis, periodontal assessment, assessment of periapical disease, endodontics, dental trauma) and surgical applications (exodontia, implant dentistry, bony pathosis, facial trauma, orthognathic surgery, TMJ) is presented [6]. In this review we briefly discuss the indications for CBCT use in dentomaxillofacial radiology and present some examples.

**Fig. 10:**
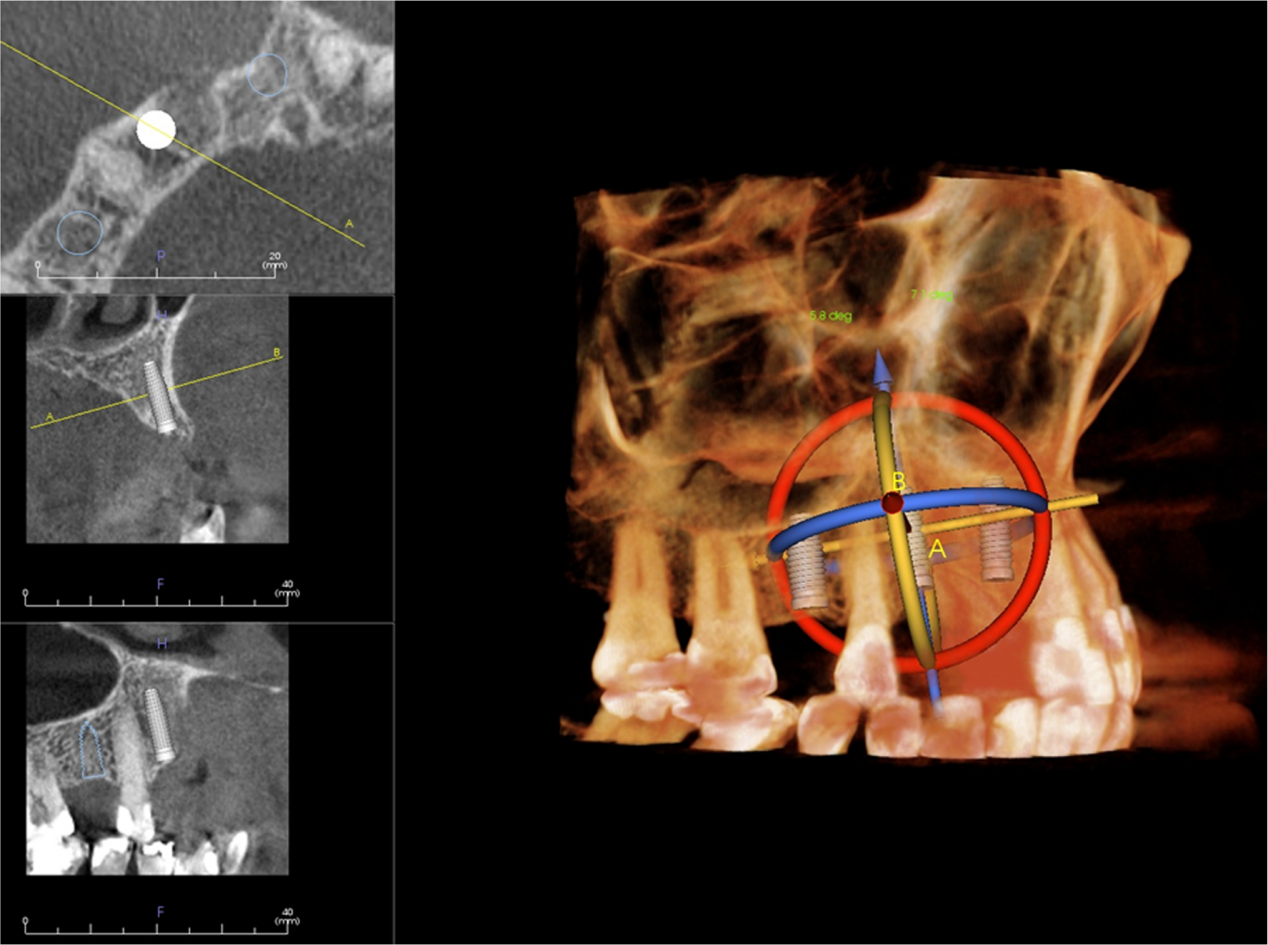
Virtual implant treatment planning with CBCT data: software can help implant treatment planning through simulation and 3D reformation. (Courtesy Jari Mauno.)

CBCT imaging is helpful in implant treatment planning. The European Association for Osseointegration (EAO) and The International Congress of Oral Implantologists (ICOI) have published their recommendations quite recently [36, 37]. If the clinical assessment of implant sites indicates that there is sufficient bone width and the conventional radiographic examination reveals the relevant anatomical boundaries and adequate bone height and space, no additional imaging is required for implant placement [36]. The literature supports the use of CBCT in dental implant treatment planning particularly in regard to linear measurements, 3D evaluation of alveolar ridge topography, proximity to vital anatomical structures and fabrication of surgical guides (Fig. 10). Areas such as CBCT-derived bone density measurements, CBCT-aided surgical navigation and postimplant CBCT artefacts need further research [37].

**Fig. 11:**
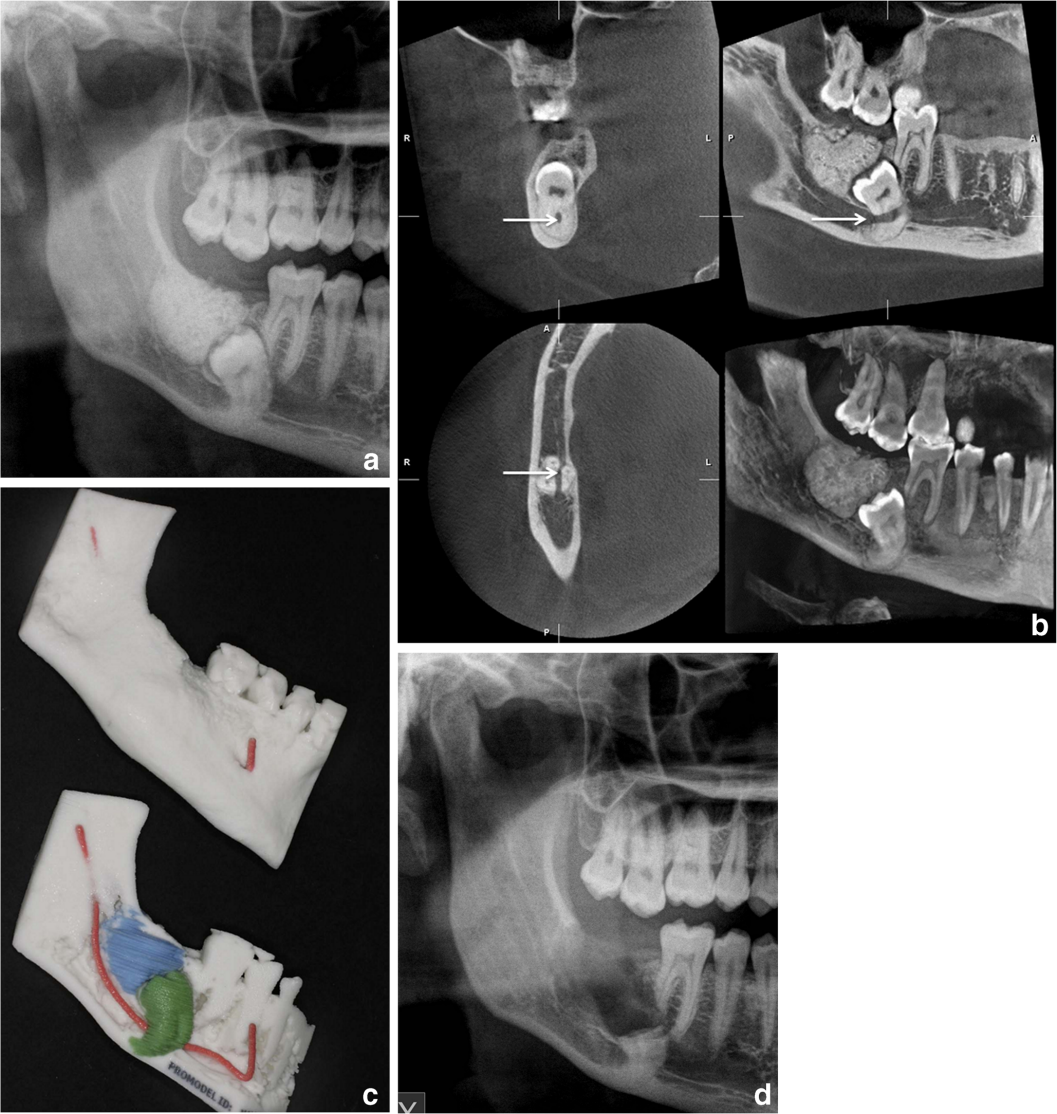
**a** Cropped PTG shows mesiocaudal dislocation of d. 47 with the root tips projecting into the lower cortex of the mandible. Distocranially to the crown of d. 47 a large complex odontoma is visible. **b** Based on the CBCT examination the mandibular canal could be located between the buccal and lingual roots (*arrows*); the root tips are in contact apically. **c** Rapid prototyping models based on the CBCT examination. **d** Postoperative cropped PTG: the roots of d. 47 were not removed in order to avoid nerve damage of the mandibular nerve bundle

**Fig. 12:**
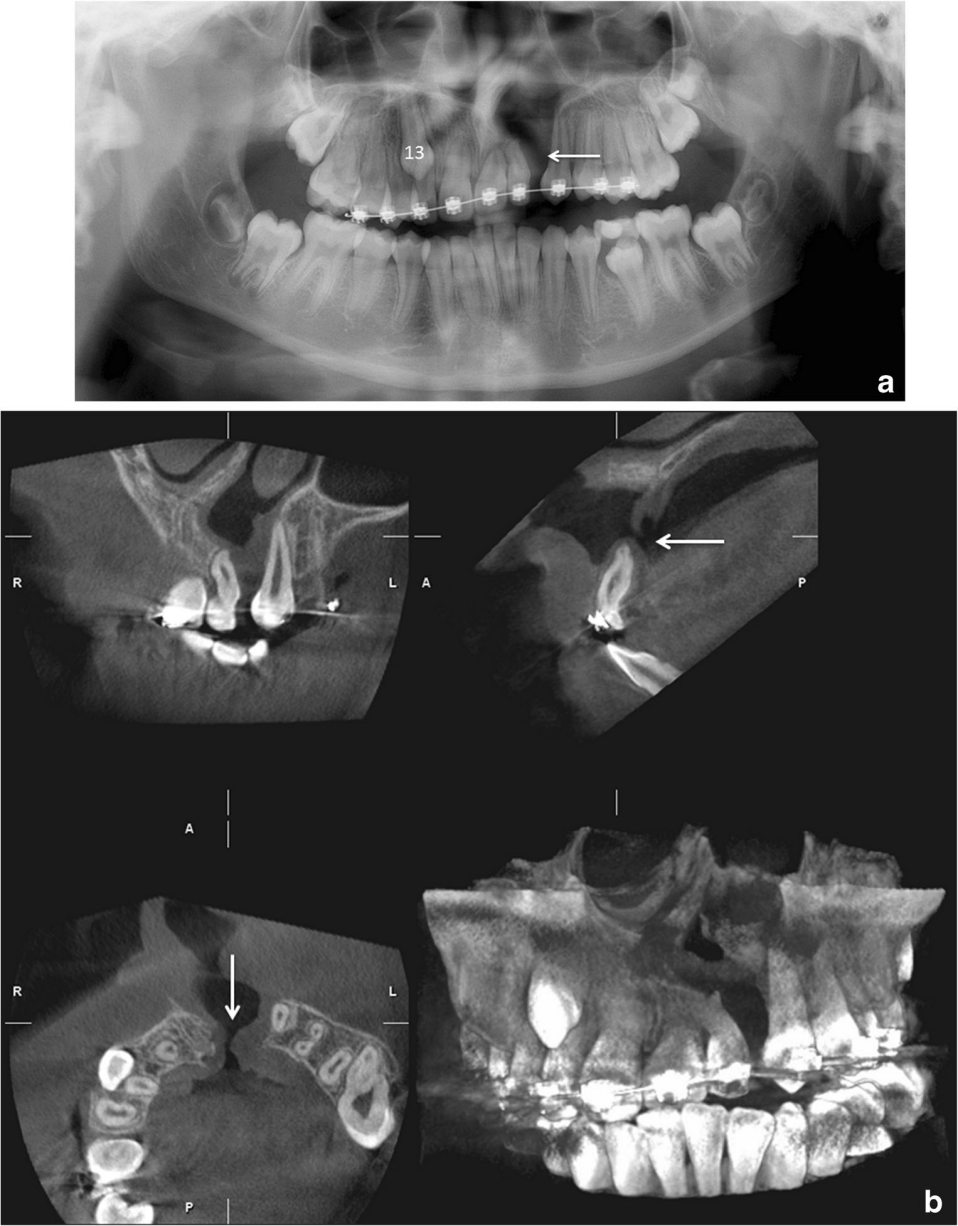
**a** A patient with a unilateral cleft lip and palate has an alveolar defect on the left side (*arrow*). A PTG image taken before secondary alveolar bone grafting (*SABG*). Note also crowding in region d. 13, which can also be easily evaluated in the CBCT examination. **b** CBCT for the treatment planning before SABG allowing evaluation of the bone defect with oronasal fistula (*arrows*)

Tooth localisation is another typical indication for CBCT imaging (Figs. 11 and 12). According to the Sedentexct guidelines [6] for the localised assessment of an impacted tooth (including consideration of resorption of an adjacent tooth) where the current imaging method of choice is MSCT, CBCT may be preferred because of the reduced radiation dose. In addition, where conventional radiographs suggest a direct interrelationship between a mandibular third molar and the mandibular canal, and when a decision to perform surgical removal has been made, CBCT may be indicated [6]. However, only very few high-evidence studies on the efficacy of CBCT for radiographic examination of mandibular third molars exist [38].

**Fig. 13:**
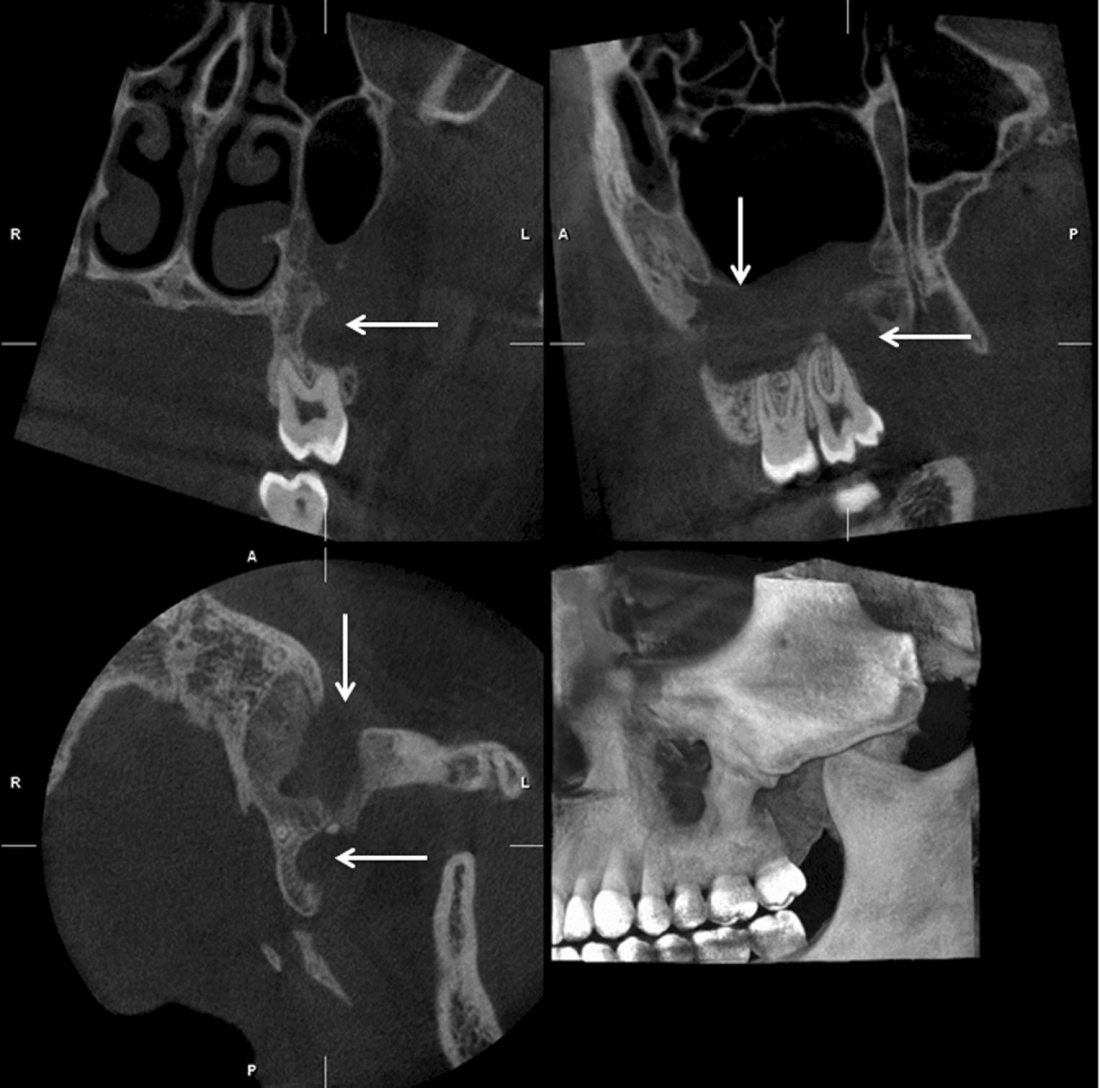
A large KCOT has been operated on in the left maxillary sinus. CBCT examination revealed a recurrence distally to d. 27, which was histopathologically proven (*horizontal arrows*). Note also the postoperative defect anteriorly and mucosal swelling in the maxillary sinus (*vertical arrows*)

CBCT also has a useful role in the assessment of bony pathosis of the jaws, for example odontogenic cysts/tumours, and it is often very helpful in their follow-up, especially in the maxillary region (Figs. 2 and 13). If soft tissue evaluation is needed MSCT or MRI is indicated. CBCT-based data sets can also be used for the fabrication of surgical 3D rapid prototyping models [39] (Fig. 11).

**Fig. 14:**
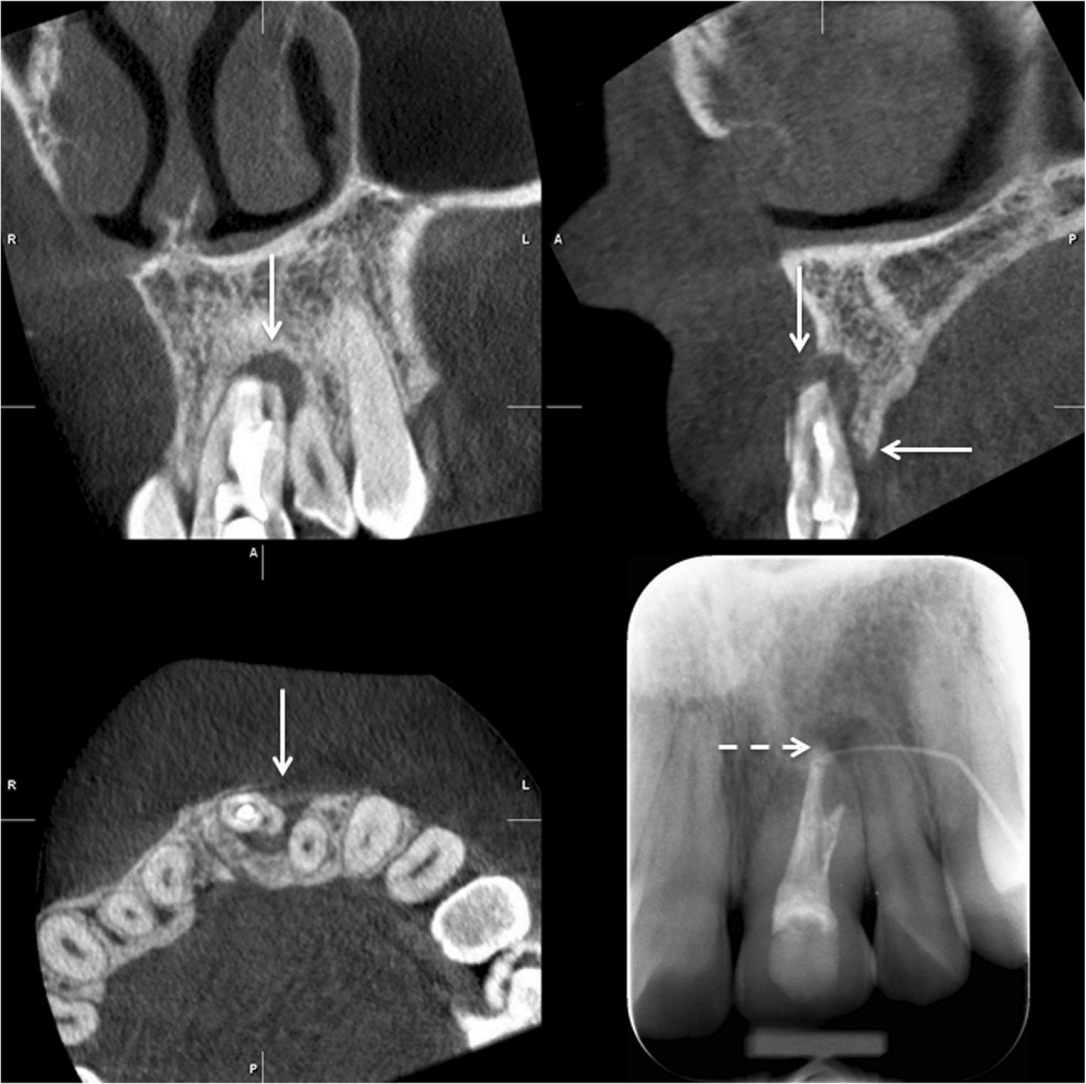
A deformed d. 21 was treated endodontically and resected several years ago. Clinically a fistula in the region d. 21 was found and fistulography with periapical radiography was done showing an apical periodontitis lesion (*lower right*). For the treatment planning a CBCT examination was done showing a large lesion indicating apical periodontitis with perforation of the labial cortex. Palatinally a vertical bone pocket is evident. Based on the CBCT findings extraction of the d. 21 was planned followed by implant treatment

**Fig. 15:**
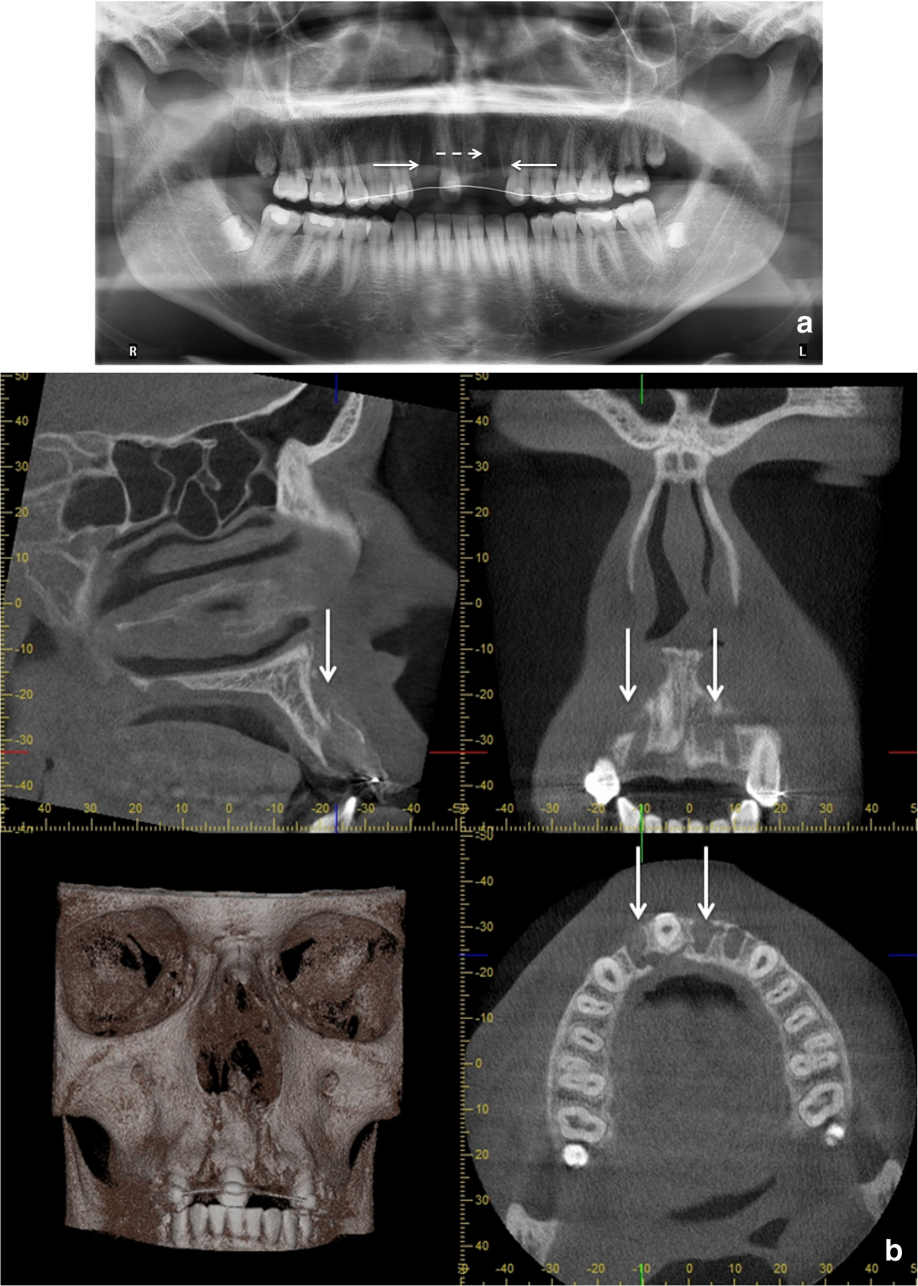
The patient had been in a motorcycle accident. (**a**) Dd. 12, 21 and 22 have been exarticulated and their root sockets are visible in the PTG (*horizontal arrows*). Alveolar fracture (*dashed arrow*) is not so easily visible in PTG in comparison to the CBCT examination where the alveolar fracture with dislocation is evident (*vertical arrows*) (**b**). (Courtesy Tapio Tammisalo.)

Limited volume, high-resolution CBCT can be used in complicated endodontic cases when conventional imaging methods provide inadequate information (Fig. 14). Mota de Almeida et al. (2014) found that CBCT has a significant impact on therapeutic decision efficacy in endodontics when used in concordance with the Sedentexct guidelines [40]. Quite recently the European Society of Endodontology (ESE) prepared a position statement concerning the use of CBCT in endodontics including dento-alveolar trauma [41]. For maxillofacial fracture assessment, where cross-sectional imaging is judged to be necessary, CBCT can be used as an alternative imaging modality to MSCT where the radiation dose has been shown to be lower and soft tissue detail is not required (Fig. 15) [6]. In the Kaeppler et al. (2014) study CBCT imaging of suspected mandibular fractures resulted in a change in the treatment plan in 9.5 % [42]. When indicated C-arm devices allow intraoperative imaging [43].

Even though there is good evidence for the accuracy of CBCT for detection of osseous abnormalities of the TMJ, no routine use of CBCT for examination of the TMJ is recommended in the absence of evidence about its impact upon treatment decisions [6]. CBCT could be considered as an alternative to MSCT if the radiation dose with CBCT is shown to be lower (Fig. 8) [6].

Cleft palate assessment is one possible application of CBCT for orthodontics and surgery. CBCT examination can be used to determine the volume of bone needed for grafting and the adequacy of the bone fill after surgery (Fig. 12). The smallest volume size compatible with the situation should be selected because of the reduced radiation dose [6]. A large FOV CBCT should not be used routinely for orthodontic diagnosis. For complex cases of skeletal abnormality, particularly those requiring combined orthodontic/surgical management, a large FOV CBCT may be justified for planning the definitive procedure, particularly where MSCT is the current imaging method of choice [6]. In a recent systematic review of CBCT applications in orthodontics, no high-quality evidence regarding the benefits of CBCT use in orthodontics was found [44].

CBCT can also be used for sinus imaging when soft tissue contrast resolution is not mandatory [45–47]. In addition, CBCT imaging can be used for 3D segmentation of the upper airway, for example in sleep apnoea patients. Because of the limited number of adequate studies, it is difficult to generate a strong conclusion regarding the current validity and reliability of CBCT-generated 3D upper airway models [48].

## Conclusion

Panoramic radiography and intraoral radiography are still the basic imaging methods in dentomaxillofacial radiology and CBCT should be used in more demanding cases. The continuously increasing research evidence will allow the indications and benefits of CBCT to be set more precisely in the future. With the CBCT technique further improvement in the image quality and lower dose to the patient can be expected.

## Abbreviations

2D: Two dimensional
3D: Three dimensional
ALARA: As low as reasonably achievable
CBCT: Cone beam computed tomography
CP: Central plane
CT: Computed tomography
DRL: Diagnostic reference level
EAO: European Association for Osseointegration
ESE: European Society of Endodontology
FDI: Federation Dentaire Internationale
FOV: Field of view
FPD: Flat panel detector
HU: Hounsfield Unit
ICOI: International Congress of Oral Implantologists
KCOT: Keratocystic odontogenic tumour
IL: Image layer
MIP: Maximum intensity projection
MRI: Magnetic resonance imaging
MSCT: Multislice computed tomography
PTG: Panoramic tomography
SABG: Secondary alveolar bone grafting
TMJ: Temporomandibular joint
